# Interleukin-15 as a Biomarker Candidate of Rheumatoid Arthritis Development

**DOI:** 10.3390/jcm9051555

**Published:** 2020-05-21

**Authors:** Weronika Kurowska, Malgorzata Przygodzka, Michal Jakubaszek, Brygida Kwiatkowska, Wlodzimierz Maslinski

**Affiliations:** 1 Department of Pathophysiology and Immunology, National Institute of Geriatrics, Rheumatology and Rehabilitation, Spartanska 1, 02-637 Warsaw, Poland; wlodzimierz.maslinski@spartanska.pl; 2 Early Arthritis Clinic, National Institute of Geriatrics, Rheumatology and Rehabilitation, Spartanska 1, 02-637 Warsaw, Poland; mprzygodzka17@gmail.com (M.P.); michal.jakubaszek@wp.pl (M.J.); kwiatkowskabrygida@gmail.com (B.K.); 3 Mazovian Center for Rheumatology and Osteoporosis, Kinowa 19a, 04-030 Warsaw, Poland

**Keywords:** undifferentiated arthritis, rheumatoid arthritis, interleukin-15, biomarker, cytokines, diagnostic of RA, RA development

## Abstract

There is a need for definite diagnosis of rheumatoid arthritis (RA) at its earliest stages of development in order to introduce early and effective treatment. Here we assessed whether serum interleukin-15 (IL-15) can serve as a new biomarker of RA development in patients with undifferentiated arthritis (UA). Interleukin-15, IgM-rheumatoid factor (RF) and anti-cyclic citrullinated peptide antibodies (anti-CCP Abs) were measured in UA patients at inclusion. Six months later, the diagnosis was re-evaluated, and statistical analysis was performed. We found that at the UA stage, IL-15 was more prevalent in patients who progressed to RA than RF or anti-CCP Abs (83.3% vs. 61.1% and 66.7%, respectively). Interleukin-15 showed higher sensitivity (77.8%) than both autoantibodies and higher specificity (80.9%) than anti-CCP Abs in identification of UA patients who developed RA. The diagnostic utility of IL-15 was comparable to that of RF (AUC: 0.814 vs. 0.750, *p* > 0.05), but higher than that of anti-CCP Abs (AUC: 0.814 vs. 0.684, *p* = 0.04). The combined use of IL-15, RF and anti-CCP Abs yielded higher diagnostic accuracy for RA than autoantibodies determination only. Our results indicate that IL-15 can be used as a biomarker of RA development in patients with UA.

## 1. Introduction

Rheumatoid arthritis (RA) is the most common inflammatory arthropathy worldwide affecting up to one percent of the adult population [1]. The disease is characterized by a symmetrical pattern of arthritis and occurrence of systemic symptoms that all lead to irreversible joint destruction, disability and premature death. There are data indicating that early aggressive treatment can be beneficial for preventing further development of the disease. These observations suggest the existence of the window of opportunity within the first few months of the disease, before joint destruction begins, for effective therapeutic intervention [2,3,4]. However, the application of such a treatment strategy is justified only if the benefit-risk ratio and cost-effectiveness balance are favorable, hence the definite diagnosis of the disease at its earliest stages of pathogenesis should be made. Unfortunately, the diagnosis of RA is often not straight forward at the early phases of disease development where clinical symptoms and currently used laboratory parameters do not allow identifying patients who will progress to RA. It was estimated that about 35–54% patients included into Large European Early Arthritis cohorts do not meet criteria for specific diseases and thus are considered as patients with undifferentiated arthritis (UA) [5]. However, as these diseases differ in the pathogenesis, the response to treatment with nonsteroidal anti-inflammatory drugs (NSAIDs) or/and glucocorticosteroids is often not satisfactory, especially in terms of hampering disease progression towards RA. For example, statistics, also from our Institute, suggest that despite this treatment about 30–40% of those patients with UA will develop RA within 6–12 months [6,7,8]. In this situation, one of the greatest challenges of rheumatology is the need for early, definite diagnosis of RA at the stage of UA. Therefore, the identification of key biomarkers that may help one recognize UA patients who will develop RA or other diseases in the near future is of particular importance.

Current progress in understanding the pathogenesis of RA implicates some candidate biomarkers of RA development. Considerable candidates are cytokines whose deregulated expression and significance in driving RA are well documented [9,10]. Importantly, blockade of biological activities of several of them, including TNF-alpha, IL-6 and IL-1 led to the development of a new class of biological drugs that are today used for the treatment of RA patients [9,10]. The role of cytokines and autoantibodies in development of RA was studied in blood samples isolated a year to five years before the disease onset. Even at this pre-RA stage, the serum levels of a large number of cytokines, chemokines, growth factors and autoantibodies were elevated in comparison to healthy people [11]. Interestingly, the pattern of these factors was similar to that observed in early RA patients. These findings were confirmed (although using partially overlapping biomarkers) in another laboratory using blood samples from a different cohort of pre-RA patients [12]. Deane [12] reported elevated levels of many immune parameters and auto-antibodies prior to the onset of RA. Our and others previous observations suggest that IL-15, one of the cytokines involved in pathogenesis of RA, could also be a marker of RA development [13,14,15]. Elevated levels of this cytokine were documented in RA and increased concentrations of IL-15 predicted severe disease course in patients with early RA or UA [13,14,15]. However, data on serum expression of IL-15 in UA patients are scarce, and the predictive and discriminative ability of IL-15 in recognizing RA has not been analyzed so far.

This study was undertaken to verify the hypothesis that, at the UA stage, IL-15 is a useful biomarker for identification of patients who will develop RA in the future.

## 2. Experimental Section

### 2.1. Patients

The study involved 65 patients with UA who had been recruited at the Early Arthritis Clinic, National Institute of Geriatrics, Rheumatology and Rehabilitation. At enrollment, patients were above 18 years old, had at least one swollen peripheral joint, and did not fulfill the American College of Rheumatology/European League Against Rheumatism Collaborative Initiative (ACR/EULAR) classification criteria for any rheumatic disease. Patients with crystal arthritis, infection symptoms, known autoimmune disease, and known cancer were excluded. Patients at the time of selection for the study were treated with nonsteroidal anti-inflammatory drugs (NSAIDs) and/or paracetamol, but not disease modifying drugs (DMARDs) and/or glucocorticosteroids. Further treatment of patients depended on the course and activity of the disease. Every individual UA patient was observed over the follow-up period of 6 months. By the end of this period, verification of diagnosis was performed and final diagnosis was established.

The written informed consent to participate in the study was obtained from every recruited patient and the study was approved by the Ethics Committee of National Institute of Geriatrics, Rheumatology and Rehabilitation, Warsaw, Poland (approval: 29/03/2012).

### 2.2. Biological Material

Venous peripheral blood was taken from UA patients at inclusion using a Vacutainer^TM^ System (BD, Plymouth, UK). Blood was processed to obtain serum samples within 1 h after collection. Serum samples were frozen at −80 °C until analysis.

### 2.3. Measurement of Autoantibodies

Autoantibodies were measured in patients’ sera as a part of routine diagnostic scheme. Rheumatoid factor (IgM–RF; RF) was detected by nephelometry using IMMAGE Immunochemistry Systems Rheumatoid Factor (Beckman Coulter, Brea, CA, USA) and anti-cyclic citrullinated peptide antibodies (anti-CCP Abs) were detected using electrochemiluminescent immunoassay (ECLIA) (Roche Diagnostics, Indianapolis, IN, USA); both according to manufacturers’ instructions. The detection level for RF was 20 IU/mL and for anti-CCP Abs 7 IU/mL.

### 2.4. Measurement of IL-15

Concentrations of IL-15 in patients’ sera were measured using enzyme-linked immunosorbent assays from RayBiotech (Peachtree Corners, GA, USA) according to the manufacturers’ instructions. The minimum detectable amount of IL-15 was 3 pg/mL.

### 2.5. Statistical Analysis

According to the Shapiro–Wilkinson test, data obtained in the study fit a non-normal distribution. Therefore, for comparison of biomarker levels in patients’ groups, the non-parametric Mann–Whitney U test was used. Correlation of biomarkers concentrations was assessed using Spearman’s rank correlation test. The discriminative ability of biomarkers in recognizing RA was determined using receiver operating characteristics (ROC) curve and area under the curve (AUC). The optimal cut-offs that best distinguished RA from non-RA patients were determined for every investigated variable at the maximum value of Youden’s index. Differences in AUC were analyzed using the z-test. *P* values below 0.05 were considered as significant. Analyses were performed using Statistica 13 software (StatSoft Polska, Kraków, Poland).

## 3. Results

### 3.1. Patient Characteristics

Forty-six women and 19 men with UA participated in the study. At enrollment, the mean age of patients was 51 ± 19.1 years. At the end of follow-up, 18 (27.7%) of the studied patients developed RA (UA→RA patients), 34 (52.3%) remained at the UA stage (UA→UA), and 13 (20%) developed another arthritic condition (UA→other): 5 patients (7.7%) osteoarthritis, 2 patients (3.1%) psoriatic arthritis, 1 patient (1.5%) reactive arthritis, 1 patient (1.5%) sarcoidosis, 2 patients (3.1%) spondyloarthropathy, 1 patient (1.5%) connective tissue disease, and 1 patient (1.5%) Sjögren Syndrome. The percentage of UA patients who experienced progression to RA was in a range of published data [6,7]. Demographical, immunological and clinical characteristics of patients grouped by the final diagnosis (UA→RA, UA→UA and UA→other) at baseline are presented in Table 1.

### 3.2. Higher Prevalence of Detectable IL-15 Levels in UA→RA Patients

Our first observation was that IL-15 was more prevalent than currently used biomarkers of RA, i.e., anti-CCP Abs and RF, in UA→RA patients. Interleukin-15 was detected in 15 (83.3%) UA→RA patients, 11 (32.4%) UA→UA patients, one patient who developed osteoarthritis, and one patient who developed reactive arthritis (Table 1). For comparison, RF was detected in 11 (61.1%) UA→RA patients, 3 (8.8%) UA→UA patients, and one patient who developed psoriatic arthritis, whereas anti-CCP Abs were detected in 12 (66.7%) UA→RA patients, 10 (29.4%) UA→UA patients, one patient who developed Sjögren Syndrome, and one patient who developed systemic connective tissue disease.

### 3.3. Higher Level of IL-15 in UA→RA Patients

**Figure 1:**
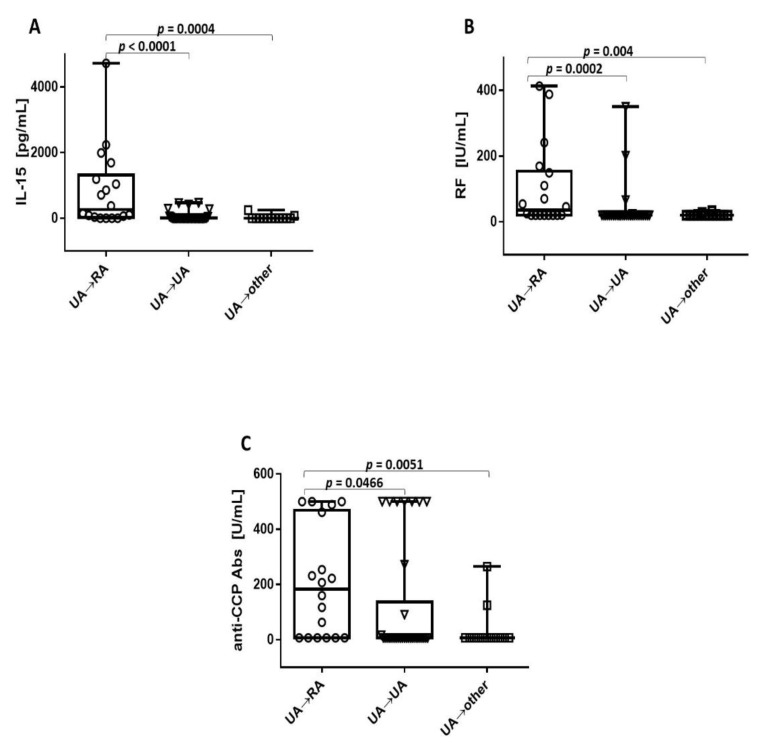
Higher levels of IL-15 in UA→RA patients. Baseline concentrations of IL-15 (**A**), RF (**B**), and anti-CCP Abs (**C**) in UA→RA patients versus UA→UA patients or patients who developed other types of arthritic conditions (UA→other). Data are presented as box plots with points, where the boxes represent the 25th to 75th percentiles, the lines within the boxes represent the median, the lines outside the boxes represent the highest and lowest values, and the point represents an individual data point.

Further analysis revealed that IL-15 levels were significantly higher in UA→RA patients than in other patient groups (Figure 1A). The median level of IL-15 in UA→RA patients was 260.8 pg/mL (range 3.3–4723 pg/mL), in UA→UA patients 3.3 pg/mL (range 3.3–472.7 pg/mL), and in UA→other patients 3.3 pg/mL (range 3.3–247.5 pg/mL). Increased levels of RF and anti-CCP Abs were also noted in UA→RA (Figure 1B and C, respectively); and, when comparing the concentrations of antibodies and IL-15 between UA→RA and UA→UA patients, the statistical significance for all parameters was obtained (*p* = 0.0466 for anti-CCP Abs, *p* = 0.0002 for RF and *p* < 0.0001 for IL-15). The median level of RF in UA→RA patients was 35.5 pg/mL (range 20–413 IU/mL), in UA→UA patients 20 IU/mL (range 20–350 IU/mL), and in UA→other patients 20 IU/mL (range 20–34,6 IU/mL) (Figure 1B). The median level of anti-CCP Abs in UA→RA patients was 183.3 IU/mL (range 7–500 IU/mL), in UA→UA patients 7 IU/mL (range 7–500 IU/mL), and in UA→other patients 7 IU/mL (range 7–265 IU/mL) (Figure 1C).

### 3.4. Expression of IL-15 Did not Overlap Entirely with RF or Anti-CCP Abs in UA→RA Patients

**Figure 2:**
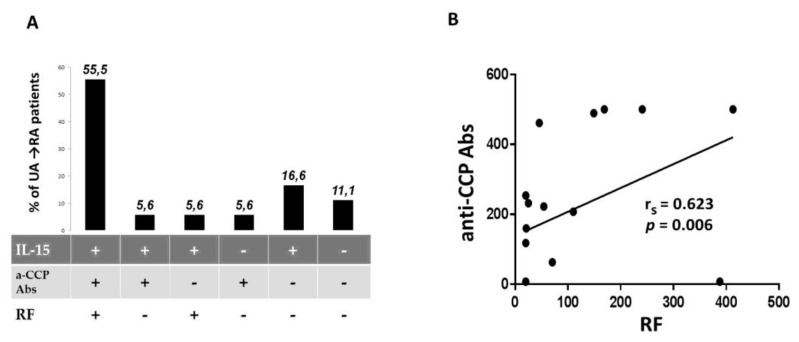
Expression of IL-15 did not overlap entirely with RF or anti-CCP Abs in UA→RA patients at baseline. (**A**) Positivity rate of biomarkers combinations in UA→RA patients. (**B**) Correlation of RF and anti-CCP Abs concentrations in UA→RA. Abbreviation: r_s_: Spearman rank correlation coefficient.

Our results indicate frequent co-occurrence of studied biomarkers in UA→RA patients at baseline (Figure 2A). Interleukin-15 together with RF and/or anti-CCP Abs was detected at the UA stage in 12 (66.7%) of those patients. Despite this phenomenon we did not observe correlation between IL-15 and RF (*p* = 0.08) or anti-CCP Ab (*p* = 0.077) concentrations. However, strong correlation between RF and anti-CCP Abs concentrations was noted (*p* = 0.006, r_s_ = 0.623) (Figure 2B). Interestingly, in some UA→RA patients (16.6%) only IL-15 was detected and none of the analyzed autoantibodies were found (Figure 2A). These data suggest that IL-15 can be, at least partially, an independent biomarker of RA development. In addition, measurement of IL-15 levels at the UA stage can help identify future RA patients who do not show anti-CCP Abs or RF in serum six months prior to diagnosis. It should be noted, however, that in the sera of two (11.1%) UA→RA patients we did not detect the biomarkers tested here (Figure 2A).

### 3.5. Interleukin-15 Shows High Diagnostic Utility in the Identification of Patients with UA who Progress to RA

**Figure 3:**
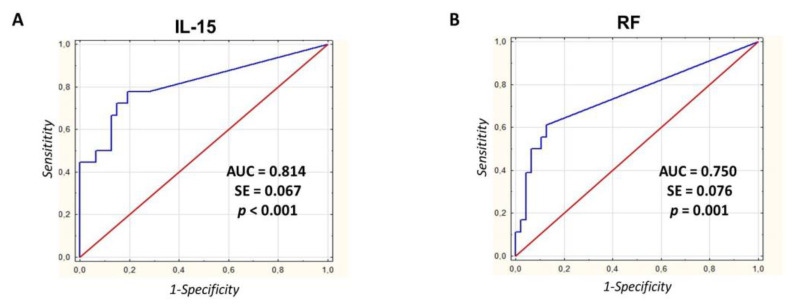

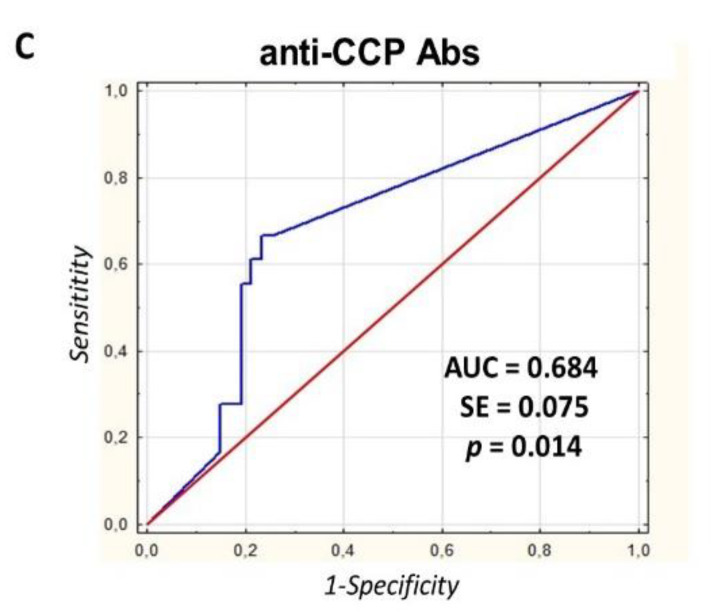
Interleukin-15 shows high diagnostic utility in the identification of patients with UA who progress to RA. Receiver operating characteristic curves (ROC) of IL-15 (**A**), RF (**B**), and anti-CCP Abs (**C**) were used in the prediction of diagnosis of RA (comparison of RA patients with non-RA patients). The curves plot the relationship between the true-positive rate (sensitivity) and the false-positive rate (1—specificity) for different cut-off levels of test positivity. Abbreviations: AUC: area under the curve; SE: standard error.

Using the area under the ROC curve (AUC) as an index of the overall discriminative ability of the test, we compared the diagnostic utilities of examined biomarkers. At the UA stage, IL-15 levels differentiated future RA patients from those who did not develop RA (non-RA patients) more accurately (AUC = 0.814, SE = 0.067, 95% CI:0.682–0.947; *p* < 0.001) than RF (AUC = 0.750, SE = 0.076, 95% CI:0.602–0.898; *p* = 0.001) or anti-CCP Abs (AUC = 0.684, SE = 0.075, 95% CI:0.538–0.830; *p* = 0.014) (Figure 3). Statistically, AUC of IL-15 was comparable with that of RF (*p* > 0.05) but higher than that of anti-CCP Abs (*p* = 0.04). Estimated by ROC curve analysis, the optimal cut-off level for IL-15 was 36.4 pg/mL with a sensitivity of 77.8% and a specificity of 80.9%. The optimal cut-offs for anti-CCP Abs and RF were set at 62.8 and 20.9 IU/mL, respectively. The sensitivity and specificity were 66.7% and 76.6% for anti-CCP Abs, and 61.1% and 87.2% for RF, respectively (Table 2).

### 3.6. Determination of IL-15 Adds a Diagnostic Value to Autoantibodies in Identifying Seropositive UA Patients who Progress to RA

We further investigated whether determination of serum IL-15 levels can improve the diagnostic performance of anti-CCP Abs and RF at the UA stage (Table 3). Adding IL-15 to RF increased the specificity (97.9% vs. 87.2%) and showed the same sensitivity (61.1%) as testing RF alone. The combination of IL-15 with anti-CCP Abs also increased specificity (93.6% vs. 76.6%), while sensitivity decreased (61.1% vs. 66.7%) compared to anti-CCP Abs alone. The combination of IL-15 with RF and anti-CCP Abs showed higher specificity (97.9% vs. 91.5%), but the same sensitivity (55.6%) compared to the combination of RF and anti-CCP Abs. Considering the overall diagnostic accuracy, the addition of IL-15 to RF or anti-CCP Abs increased diagnostic performance when compared with those for RF or ani-CCP Abs alone (87.7 vs. 80.0 and 84.6 vs. 73.8, respectively). By determining IL-15 and both of these autoantibodies in patient’s sera, diagnostic accuracy was also higher than that when using the combination of RF and anti-CCP Abs (86.2 vs. 81.5).

## 4. Discussion

Recent findings led to changes in RA treatment recommendations with emphasis on early application of aggressive therapy [16]. However, pathological events that drive the transition from undifferentiated state to RA are still not well understood. A scarcity of biomarkers that reflect these pathological processes make it difficult to define diagnostic criteria for RA at early phases of disease development. Even anti-CCP Abs approved in 2010 by experts from ACR and EULAR as a diagnostic criterion for RA [17] do not allow for definite diagnosis at early stages of the disease [18,19]. Recommended testing of anti-CCP Abs and RF levels can be insufficient for identification of patients who will progress to RA in the near future, as these autoantibodies can be present over years in asymptomatic subjects [18]. In addition, RA is considered a heterogenic disease resulting from many immunological abnormalities. Thus, for better treatment tailoring, the stratification of patients based on mechanism of disease rather than by clinical phenotype should be performed [20]. In this context, biomarkers that reflect those diverse molecular disturbances in the early stages of disease are urgently needed. Interleukin-15 seems to be a good biomarker candidate in this respect. It is innate response cytokine that mediates pleiotropic effector functions in RA. Increased levels of IL-15 were detected in serum, synovial fluid and bone marrow of RA patients compared to patients with other inflammatory arthropathies and/or osteoarthritis [21,22,23,24,25]. In established RA, IL-15 promoted TNF-α and IL-17 production, activation of T lymphocytes and stimulation of osteoclastogenesis [25,26,27]. Genetic variants in IL-15 associated with progression of joint destruction in RA [28]. Neutralization of this cytokine diminished arthritis in animal models and patients with RA [29], and agents blocking IL-15 were shown to be effective in clinical trials [30,31]. Interestingly, our and other observations indicate that IL-15 can play a role in early phases of RA development. In patients with early RA the levels of IL-15 were shown to be elevated and predicted severe disease course [13,14]. Increased serum concentration of IL-15 was also documented in pre-clinical RA [12].

The results of this study indicate that IL-15 can be detected with high frequency in the sera of patients with UA, who developed RA in the next six months. This finding confirmed our recent pilot observations [15]. In those patients, elevated concentrations of IL-15 were also noted. Interestingly, the increase in IL-15 level in UA patients who experienced progression to RA was more pronounced than the increase in RF or anti-CCP Abs. This observation suggests the involvement of IL-15 in the pathogenesis of RA in the early stages of the disease and implies that blocking IL-15 activity at this time may have therapeutic potential.

Our study revealed detectable amounts of IL-15 (>3 pg/mL) in some patients remaining in the UA stage, which might diminish the strength of the test we used for evaluating IL-15 as a biomarker for RA development. However, despite this phenomenon, analysis of the diagnostic utility of tested variables at the UA stage revealed that the overall accuracy of IL-15 in predicting RA was even higher than those of anti-CCP Abs or RF. Furthermore, it is also possible that this group of UA patients with elevated IL-15 concentration will develop RA in the near future. Theoretically, this could further improve the diagnostic performance of IL-15, because this cytokine was the only one of the tested biomarkers detected in 45.5% of patients still at the UA stage.

We established cut-off levels of IL-15, RF and anti-CCP Abs that best distinguished UA patients who progressed to RA from whose who did not develop RA within 6 months of follow-up. Based on these parameters we found that the sensitivity and specificity of IL-15 for diagnosis of RA was higher or at least comparable to those observed for anti-CCP Abs or RF. Worthy of notice, 16.7% seronegative patients who progressed to RA could be identified at the UA stage by their expression of IL-15. These observations point to the potential of IL-15 in improving RA diagnostics, particularly in early seronegative arthritis.

Moreover, the detected levels of IL-15 did not overlap simply with anti-CCP Abs and RF. We did not find correlation between concentrations of IL-15 and any of tested autoantibodies in UA patients who progressed to RA. However, concentrations of RF and anti-CCP Abs correlated significantly in those individuals. This latter observation is consistent with the data showing that concentrations of both these autoantibodies increase up to the onset of the disease [32,33] and the combined presence of higher concentrations of both anti-CCP Abs and RF yields a higher score for the diagnosis of RA [17]. In our study, IL-15 levels also did not correlate with CRP and ESR values. These results suggest that IL-15 can be at least a partially independent (from currently used indicators) biomarker of RA development.

When a combination of biomarkers was used for estimation of diagnostic accuracy, the addition of IL-15 to any of tested autoantibody or to combination of both autoantibodies was superior to those for RF or ani-CCP Abs alone, or the combination of both autoantibodies. Therefore, serum IL-15 evaluation, along with currently used autoantibodies, may be beneficial in identifying seropositive UA patients who progress to RA.

In studied patients there were also two individuals who developed RA and did not display any of investigated serum biomarkers (IL-15, RF or anti-CCP Abs). This can mirror the complexity of pathogenesis of RA and points to the need for further research.

Our study is the first to our knowledge fully dedicated to investigating IL-15 as a predictive biomarker of RA development in UA patients. The limitation of our study is the follow-up time of six months and a relatively small number of subjects studied. Therefore, our findings should be further validated in other medical centers and larger populations of patients. In addition, we did not check IL-15 levels during 6 months of follow-up and after the time the diagnosis was established. The kinetics of IL-15 concentration remains an interesting issue to study in future research. To this end, multiplex assays with high sensitivity of cytokine detection can be used [34].

To summarize, our results indicate that IL-15 can play a role in the early stages of RA development as it can be detected with a high prevalence and at elevated concentrations in serum of UA patients who converted to RA during six months of follow-up. Moreover, IL-15 can represent a relevant, independent biomarker candidate for prediction of the development of RA. Testing IL-15 levels can help to identify future seronegative RA patients in the early phase of disease pathogenesis. Measuring easily accessible circulating IL-15 can improve diagnostics of RA at the UA stage and allow introduction of more efficient, personalized treatment.

## Figures and Tables

**Table 1:** Basic characteristics of patients grouped by the final diagnosis.

Parameter	Group of Patients		
	UA→RA	UA→non-RA ^1^	
		UA→UA	UA→other
Age, mean ± SD (years)	50 ± 20.4	51.4 ± 17.7	
		49.6 ± 18.6	56.1 ± 15.6
Sex (%)	13 females (72.2)	33 females (70.2)	
		14 males	
	5 males		
		23 females (67.6)	10 females (76.9)
		11 males	3 males
CRP [mg/L] (range)	17.5 (4–70)	8 (1–64) ^#^	
		10 (2–49)	6.5 (1–64)
ESR [mm/h] (range)	33 (7–110)	16 (3–71) *	
		18 (4–71) **	12 (3–52) ***
IL-15 positivity ^2^ (%)	15 (83.3)	13 (27.7)	
		11 (32.4)	2 (15.4)
RF positivity (%)	11 (61.1)	4 (8.5)	
		3 (8.8)	1 (7.7)
anti-CCP Abs positivity (%)	12 (66.7)	12 (25.5)	
		10 (29.4)	2 (15.4)
Swollen joints count (range)	4 (1–12)	2 (1–12)	
		2 (1–9)	1 (1–12)

**^1^** UA→non-RA patients are those who at the end of follow-up remained at the UA stage (UA→UA) and patients who developed other arthritic conditions (UA→other). **^2^** Defined as IL-15 concentration above the detection limit of 3 pg/mL. The median values of parameters are shown unless indicated otherwise. Statistical difference comparing to UA→RA patients’ group is shown: ^#^ *p* = 0.043, * *p* = 0.005, ** *p* = 0.006, *** *p* = 0.002. Abbreviations: CRP: C-reactive protein; ESR: erythrocyte sedimentation rate; SD: standard deviation.

**Table 2:** Cut-off levels, sensitivities and specificities of biomarkers for development of RA in patients with UA.

Biomarker	Optimal Cut-Off	Sensitivity (%)	Specificity (%)
IL-15	36.4 pg/mL	77.8	80.9
anti-CCP Abs	62.8 IU/mL	66.7	76.6
RF	20.9 IU/mL	61.1	87.2

Areas under the ROC curves were used to compute sensitivity and specificity of biomarkers and to determine the optimal cut-offs.

**Table 3:** Diagnostic values of biomarkers for RA.

Combination of Biomarkers	Sensitivity (%)	Specificity (%)	PPV	NPV	Diagnostic Accuracy
anti-CCP Abs	66.7	76.6	52.2	85.7	73.8
RF	61.1	87.2	64.7	85.4	80.0
IL-15	77.8	80.9	60.9	90.5	80.0
IL-15 or anti-CCP Abs	83.3	63.8	46.9	90.9	69.2
IL-15 or RF	77.8	70.2	50.0	89.2	72.3
IL-15 and anti-CCP Abs	61.1	93.6	78.6	86.3	84.6
IL-15 and RF	61.1	97.9	91.7	86.8	87.7
IL-15 and anti-CCP Abs and RF	55.6	97.9	90.9	85.2	86.2
anti-CCP Abs and RF	55.6	91.5	71.4	84.3	81.5
anti-CCP Abs or RF	72.2	72.3	50.0	87.2	72.3
IL-15 or anti-CCP Abs or RF	83.3	59.6	44.1	90.3	66.2

Abbreviations: PPV: positive predictive value; NPV: negative predictive value.
